# Correction

**DOI:** 10.1080/28338073.2025.2454117

**Published:** 2025-01-21

**Article title**: Revolutionizing Diabetic Retinopathy Screening: Integrating AI-Based Retinal Imaging in Primary Care

**Authors**: Dean Beals, Lesley Simon, Faith Rogers and Stan Pogroszewski

**Journal**: *Journal of CME*

**Bibliometrics**: Volume 14, Number 01, pages 1-7

**DOI**: https://doi.org/10.1080/28338073.2024.2437294

It has been noted by the corresponding author that the name “Dale Kummerle” was inadvertently included in author list of the article. The correct authorship of the article should be “Dean Beals, Lesley Simon, Faith Rogers, and Stan Pogroszewski.” This correction does not affect the description, interpretation, or original conclusions of the article. The authors apologize for any inconvenience caused. The authors gratefully acknowledge the contributions of Dale Kummerle of KH Consultancy in developing, preparing, and writing this manuscript.

